# The MyToolbox EU–China Partnership—Progress and Future Directions in Mycotoxin Research and Management

**DOI:** 10.3390/toxins12110712

**Published:** 2020-11-11

**Authors:** John Leslie, Birgit Poschmaier, Hans van Egmond, Alexandra Malachová, Monique de Nijs, Ferenc Bagi, Jing Zhou, Zhen Jin, Songxue Wang, Michele Suman, Gerd Schatzmayr, Rudolf Krska

**Affiliations:** 1 Department of Plant Pathology, Throckmorton Plant Sciences Center, 1712 Claflin Avenue, Kansas State University, Manhattan, KS 66506, USA; jfl@ksu.edu; 2 Department of Agrobiotechnology (IFA-Tulln), Institute of Bioanalytics and Agro-Metabolomics, University of Natural Resources and Life Sciences, Vienna (BOKU), Konrad-Lorenz-Str. 20, 3430 Tulln an der Donau, Austria; birgit.poschmaier@boku.ac.at; 3 Watermoleneiland 15, 7241 VS Lochem, The Netherlands; hp.van.egmond@planet.nl; 4 FFoQSI—Austrian Competence Centre for Feed and Food Quality, Safety & Innovation, Head Office: FFoQSI GmbH, Technopark 1C, 3430 Tulln an der Donau, Austria; alexandra.malachova@ffoqsi.at; 5 Wageningen Food Safety Research, Akkermaalsbos 2, 6708 Wageningen, The Netherlands; Monique.denijs@wur.nl; 6 Faculty of Agriculture, University of Novi Sad, Dositeja Obradovica 8, 21000 Novi Sad, Serbia; bagifer@polj.uns.ac.rs; 7 Romer Labs China Ltd., Jia Tai International Mansion, 41 East 4th Ring Middle Road, Chaoyang District, Beijing 100025, China; zhou.jing@romerlabs.com (J.Z.); jane.jin@romerlabs.com (Z.J.); 8 Institute of Cereals and Oils Quality and Safety, Academy of National Food and Strategic Reserves Administration, 23 Yongwang Ave., Daxing District, Beijing 102600, China; wsx@ags.ac.cn; 9 BARILLA S.p.A., Food Chemistry and Safety Research, Barilla Research Labs, Via Mantova 166, 43 122 Parma, Italy; Michele.Suman@barilla.com; 10 BIOMIN Research Center, BIOMIN Holding GmbH, Technopark 1, 3430 Tulln an der Donau, Austria; gerd.schatzmayr@biomin.net; 11 Institute for Global Food Security, School of Biological Sciences, Queen’s University Belfast, University Road, Belfast BT7 1NN, UK

**Keywords:** mycotoxins, forecasting models, silo management, detoxification, biocontrol, bioethanol

## Abstract

Affordable and practical tools for farmers and food processors along the chain are required to efficiently reduce the risk of mycotoxin contamination of crops, feeds and foods. Developing new tools and enhancing existing ones was the mission of MyToolBox—a four-year EU-project that included important Chinese partners and joint research efforts. To identify future directions in mycotoxin research and management in China and their role in China–EU relations, a unique stakeholder workshop including group discussions was organized in Beijing. Six related topics: biocontrol, forecasting, sampling and analysis, silo management, detoxification, and the development of safe use options for contaminated materials were covered. The discussions clearly identified a critical need for smart, integrated strategies to address mycotoxin issues to attain safer food and feed, and to minimize losses and export rejections. Managing data on when, where and the size of mycotoxin contamination events and identifying the institution(s) to manage them are complex issues in China. Studies of microbes and novel, genetically-altered enzymes to limit pre-harvest contamination and to manage post-harvest product detoxification and alternate uses of contaminated materials are in the early stages in China. Further efforts are needed to increase the visibility of mycotoxin problems beyond the scientific and research communities.

## 1. Introduction

Up to 80% of food items of plant origin worldwide are estimated to be contaminated with mycotoxins, toxic secondary metabolites of fungi, at levels above the limit of detection (LOD) [1]. Mycotoxins threaten the health and productivity of humans and domesticated animals through dietary exposure at both acute and sub-acute contamination levels in the diet [2,3]. Many countries regulate the levels of mycotoxins allowed in imported goods, and mycotoxins are becoming an important non-tariff trade barrier [4]. Climate change will alter the distribution of mycotoxin producing fungi, including both *Aspergillus* and *Fusarium* spp. For example, hot, dry conditions that exacerbate plant stress will increase the contamination of food and feed with carcinogenic aflatoxins [5]. Cumulative exposure to mycotoxins can be reduced by careful management of these natural toxins across the food and feed production chains. Mitigation strategies developed in Europe, China and other parts of the world have helped to reduce the absolute number of highly contaminated batches of raw food and feedstuffs in Europe [1].

Recently, two large four-year EU-projects with Chinese participation MyToolBox [6] and MycoKey [7], focused on reducing mycotoxin contamination, have been successfully completed. The goal of the MyToolBox project [8] was to develop an integrated, user-friendly e-platform for managing mycotoxins by stakeholders along global food and feed chains [9]. The e-platform contains information on management practices for mitigating mycotoxin contamination in both pre- and post-harvest settings and for safe-use options, with an on-line decision support tool based on predictive models to assist participants, as needed. The project applied a multi-disciplinary approach to the problem and engaged partners who were academics, industry scientists, IT specialists, policy makers and potential end-users. This diversity was reflected in the consortium that implemented the project, which consisted of 23 organizations based in 10 European countries and China [7].

China is a global leader in the production of maize, peanuts and wheat for human food and animal feed. These crops are susceptible to colonization by mycotoxin-producing fungi and to mycotoxin contamination, sometimes at quite high levels. A ten-year survey of feed samples showed that more than 20% of samples from the Far East (China, Japan and South Korea), exceeded the maximum EU guidance levels for deoxynivalenol and zearalenone [10]. Hence, raw materials in the Chinese market contaminated with mycotoxins at levels that exceed EU regulatory limits are expected. The occurrence of aflatoxins and in particular aflatoxin B1 in the peanut supply chain poses a clear risk for European [11] and Chinese consumers. Based on data from the Rapid Alert System for Food and Feed (RASFF), mycotoxin contamination exceeding the maximum allowable limit is the most common reason for rejection of food products at the outer European Economic Area (EEA) borders. For example, over the last five years RASFF reported more than 300 alerts of aflatoxin levels exceeding legal limits in peanuts imported from China [12]. For the major mycotoxins, detailed limits for food and feed are in place in China [13]. In 2017, China released the National Food Safety Standard for Maximum Levels of aflatoxin B1, aflatoxin M1, deoxynivalenol, patulin, ochratoxin A and zearalenone in foods (GB 2761-2017) as an update of the standard GB2761-2011 [14]. Worth noticing, is that these limits correspond with the maximum levels established in the EU for most of the commodities listed. Exchange of expertise between the EU and China enables the sharing of experiences, expansion of scientific interaction networks, and synergistic solutions to mycotoxin contamination problems of interest to all parties.

A significant portion of the MyToolBox project focused on personal interactions between EU scientists and their Chinese counterparts at three Chinese universities and research institutes, and broader collaborations that went beyond the formal relationships. A major MyToolBox goal was to strengthen international cooperation in research and innovation for mycotoxins and to invigorate ties between China and Europe in the area of mycotoxin reduction.

In Europe, MyToolBox pre-harvest studies focused on alternative plant protection products and soil treatments in northwestern Europe to mitigate *Fusarium* infection [15]. Farming systems, including *Aspergillus*-resistant maize hybrids, were tested in southeastern Europe. Joint EU–China biocontrol projects focusing on the identification of new biocontrol agents (e.g., atoxigenic *Aspergillus flavus*) were developed and the results implemented to reduce aflatoxin contamination in southeast Europe (maize) and in China (peanuts). MyToolBox post-harvest studies evaluated silo management practices for wheat storage in Italy and peanut storage in China and developed CO_2_ respiration models that enabled the design and production of improved sensors for silo monitoring [16,17,18]. Evaluation of innovative milling techniques and the effects of thermal processing on wheat and wheat-based products, provided evidence for a reduction in deoxynivalenol contamination in the final products [19]. Testing for efficient safe-use options included the use of genetically altered enzymes (FUMzyme^®^) to minimize mycotoxin levels in Distillers Dried Grains with Solubles (DDGS), a protein rich by-product produced during bioethanol fermentation. The same type of enzyme, in addition to specific toxin binders, were used as feed additives in China to reduce exposure of swine to fumonisins (FUMzyme^®^) and of dairy cattle to aflatoxins (Bentonite) [20]. As a result, the EU–China partnership within MyToolBox contributed to the standard setting process for authorization of mycotoxin-detoxifying feed additives in China, with current EU guidelines for the registration of detoxifying feed additives serving as starting points for writing similar Chinese legislation. The EU–China partnership enabled the collection and analysis of data on mycotoxin contamination in winter wheat from various parts of Europe and China with a validated analytical method. Based on these collection data, contamination prediction models were customized for different climatic regions [21]. The customized prediction models are available through the e-tool [9] and can assist farmers in identifying potential risks to their crops.

While scientific publications are valuable for defining the status of, and progress in, a specific field of research, they are less useful for projecting where a field is going. To help identify future directions, MyToolBox participants, including the Chinese Academy of National Food and Strategic Reserves Administration in cooperation with Romer Labs, organized a Stakeholder Workshop on Strategies for Effective Mycotoxin Management in China. The workshop was held in Beijing from 16–17 April 2019. The program had six major sections: Biocontrol, Forecasting, Sampling and Analysis, Silo Management, Detoxification, and Safe Use Options, each with Chinese and European presenters in formal meeting presentations and stakeholders in forward-looking discussion sessions. The discussion sessions were run as Nominal Group style roundtable discussions at the end of each day following the presentations [22]. This style of group discussion has been used previously to set goals for research in mycotoxin control [23,24] and sorghum and millet improvement in Africa [25]. In Beijing, six simultaneous discussion sections were formed—one for each of the three subject areas in which presentations were made on each of the two days. The goal was to identify areas of future research and collaboration, i.e., how to build on the significant progress reported in the presentations in terms of work needed in China and in terms of EU–China interactions.

## 2. Results

### 2.1. Biocontrol

In China, there currently are three main issues with biocontrol of mycotoxin contamination (Table 1). Specifically, (i) application and commercial use of biocontrol agents, (ii) identification and deployment of organisms that can directly degrade mycotoxins, and (iii) development of new biocontrol agents.

Application and commercial use issues have to do with safety, cost, and how to evaluate efficacy (responses 1, 3 and 4). These issues are common to biological control studies globally and the issues will be if/how Chinese conditions differ from those found elsewhere. Studies of these problems would be fruitful grounds for future international collaborations.

The direct degradation of toxins by biocontrol organisms is a second important point (responses 2 and 5) and not as heavily researched. Certainly, microorganisms are degrading mycotoxins under field conditions [26], but successfully harnessing these microbes to increase food and feed safety is at an early stage. Finally, there are efforts focused on developing new biocontrols and biocontrol agents (responses 6–9). These efforts range from developing criteria to determine when an emerging technology is ready for application, determining how to recover and identify potential biocontrol agents, and identifying biocontrols for specific Chinese applications.

MyToolBox and its successors can enhance ongoing biocontrol work in China (Table 2) in several ways. The most prominent is to advance research to address particular problems (responses 1, 6 and 7), e.g., climate change, drug/fungicide resistance, and a broader spectrum of mycotoxins and associated toxin-producing fungi. A second action is to continue and expand EU/China collaborative activities (responses 2, 3 and 9). These interactions could be through bilateral institutional agreements or through multi-institutional platforms such as MyToolBox, with work on pre-commercial opportunities viewed specifically as important. Finally, the deployment of MyToolBox products and tools among Chinese farmers (responses 4, 5 and 8), is important to assist in increasing productivity. There were questions about the cost to use such tools. Pricing will depend on government funding to support adaptation to Chinese markets, crops and conditions and to maintain and update the software as additional information becomes available.

### 2.2. Forecasting

The responses provided by this discussion group were not reported in the requested format. Only the compiled ratings were reported on the flip chart and the top five responses were given with a 1–5 ordering (5 being the highest). Neither the weighted score for each response nor the number of participants ranking a response in their personal top 5 were recorded on the flipchart. Responses not included in the top 5 are not presented in any particular order and are simply listed as unranked. This format provided information for the final plenary meeting session but did not provide more detailed information on the additional responses included in the tables for the other sessions.

In China, the current issues with the forecasting of mycotoxins (Table 3) fall into three classes: (i) implementation of forecasting technology, (ii) developing new and improving existing forecasting technology, and (iii) ensuring that farmers and others understand the risks identified. There are significant issues surrounding the implementation of any forecasting system (responses 1, 2 and 4) that occupy three of the top five slots on the list. The roles of universities, government, research institutes and the private sector in developing the forecasts and in sending alerts remain to be worked out, and the responsibility for making production recommendations based on projected problems assigned. Educating farmers, traders and others with roles along the various food chains in the value, interpretation and utility of the forecasts also is essential to their long-term success as a crop management tool. Increasing the technology and infrastructure for successful forecasting models requires careful design—which production chains and how much of the chain, and when and how potential warnings will be distributed. Big data concepts and technologies are needed to successfully implement these systems and problems related to pollution and imperfections in the data must be overcome to generate usable models (responses 3, 5, 6, 7 and 9). Ensuring that farmers, traders and others with roles along the various food chains understand the risks posed by mycotoxin contamination is essential for the forecasts provided to have practical value (response 8). Without this fundamental understanding, results from forecasting models, no matter how accurate, will have limited practical value. Finally protocols for data collection, disclosure and sharing must be developed and implemented in a manner that avoids stigmatization of individual farms, crops and production regions.

MyToolBox and its successors can enhance ongoing forecasting work on mycotoxin contamination in crops in China (Table 4) in several ways. Most prominently the models being used need to be expanded and tailored to the Chinese situation (responses 3–5 and 8–10). Incorporating characteristics and conditions peculiar to China into existing models and comparing multiple forecast models ensured that an optimal one has been selected for use. Additional topics that could be incorporated into the models are the number of crops and mycotoxins monitored, post-harvest conditions (and warnings), economic variables, and the impact of biological/chemical protection of the host. There was a perceived need to encourage broader participation (responses 2, 6, 7 and 11) by focusing on key farms and engaging larger private sector stakeholders more effectively. Establishing continuing MyToolBox contacts in China to support forecasting tools and obtaining an official endorsement for them also would increase the utilization of existing and newly developed models. Finally, uses for the data collected beyond just mycotoxin forecasting, e.g., disease and yield forecasts and the impact of climate change, should be considered, and protocols for sharing the data underlying the forecasting models on mycotoxin contamination need to be developed (responses 1 and 12).

### 2.3. Sampling and Analysis

In China, the current issues with sampling and detection of mycotoxins (Table 5) are focused on ensuring available protocols, plans and methods are adequately implemented. For sampling, a major concern is that those performing the sampling and inspection are adequately trained (responses 2 and 11). With the samples themselves one set of issues revolves around the best way to collect them at a minimal cost in a manageable volume with the least disturbance to the original material (3, 10, 11 and 13). A second set of issues revolving around samples is sample quality, i.e., how to transfer and store them once they are collected, how to enable consistent representative sampling, and how to manage sample sets following detoxification procedures (responses 6, 8 and 12). A third concern is the analysis of the samples, as detection protocols focus on methods of pretreatment, speed and matrix specificity (responses 5, 9 and 12–14). Analytical protocols also were important with test repeatability at the top followed by better defined limits of detection (LODs) and limits of quantification (LOQs) for standard protocols and equipment, and the availability of certified standard materials.

MyToolBox and its successors can enhance ongoing work on sampling and analysis of mycotoxin contamination of raw materials, feed and food in China (Table 6) in several ways that parallel the ongoing efforts. Sampling methodology remains a major concern with a need to develop better sampling plans and to develop strategies for managing materials originating from areas with historically different levels of mycotoxin contamination (responses 2, 3, 7 and 12). Sample management also is an issue with traceability and shipping cross-cutting issues, and the ability to address particular matrices and other variables, e.g., host line/variety/hybrid, that could have important indirect effects (responses 1, 4, 8 and 11). With regard to detection, the requests were for faster analyses and for increased capacity through shared models and more extensive collaboration with the private sector (responses 5, 6, 9 and 10).

### 2.4. Silo Management

In China, the current issues with Post-Harvest/Silo management of mycotoxins (Table 7) are divided amongst pre-storage issues, silo equipment, design and management, and quality control of the material being stored. Paying close attention to pre-storage issues can significantly lessen problems once the crop is in storage. Items of concern here were: regional differences in grain quality, separating higher quality material from lower quality material, treating material to reduce mold and insect growth, and ensuring that the crop remains safe during transport to/from the storage facility (responses 8 and 10–12). Storage silos were the second major area of concern—with both their abilities and the processes used to manage them drawing attention. Two abilities of particular importance were the equipping of silos with sensors that could monitor the status of stored grain and the ability to treat grain with fungicides and/or insecticides (responses 3 and 11). Equally as important as the silo infrastructure were the silo management strategies (responses 4, 5 and 7). Standardization of management practices and developing and implementing Good Storage Practices is very important. These practices include efficacious drying and maintenance of appropriate storage conditions. Quality control is critical and requires appropriate equipment in the silo to monitor crop status in real-time, with appropriate software to interpret the data received from the sensors, and routine samples that are tested to ensure that quality is being maintained (responses 1–3 and 12). Finally, if problems are identified, e.g., insect damage, excess humidity, fungal growth or mycotoxin production, then the ability to deal with the problem also was essential (responses 6, 9 and 13).

MyToolBox and its successors can enhance ongoing work on post-harvest/silo management of mycotoxin contamination in China (Table 8) in several ways that complement and extend efforts already in progress. Expressed interests were for broader post-harvest issues that went beyond, but included, silo management including managing the process, obtaining additional training and the participation of China in international cooperation dealing with the mitigation of mycotoxins. Harmonizing Chinese and EU mycotoxin regulations was important to ensure continued or increased EU/Chinese trade (response 6). Adoption of the Farm to Fork concept provides a framework for food safety and production that encompasses the entire food chain and underscores the idea that what happens at one stage can impact other stages and is not a stand-alone event (response 9). Developing and sharing models of the post-harvest process to identify critical factors and combinations thereof was the most important of the projected future activities (responses 1, 2 and 8). A close second in significance was work on developing and defining post-harvest management systems, and especially developing standard ways to deal with typical problems, such as insect damage, mycotoxin contamination, or grain of inconsistent quality (responses 3–5 and 7). Finally, there was a request for continued training in mycotoxin analysis and the provision of advice based on analytical results (responses 10 and 11).

### 2.5. Detoxification

In China, the current issues with detoxification of mycotoxin-contaminated materials (Table 9) are with the process(es), their development and the quality of the detoxified product. Although not stated explicitly, the discussion was more attentive to biological detoxification agents, and less attentive to physical and chemical detoxification agents. A number of participants were unfamiliar with possible detoxification processes as indicated by queries about whether the process was allowed in China, the scope of use for the products, and the identification of companies that produce usable products (responses 11–13). Four of the top five responses (1–3 and 5) focused on the application of detoxification processes, especially the use of microorganisms and enzymes. Safety, efficiency, efficacy, reproducibility, risk of reversibility, cost, and conditions for use and application also were identified as concerns. There were questions about how the detoxification products were developed (responses 4 and 9) and about the properties of the agents themselves—adsorptive properties (including potentially negative effects from binding desirable nutrients), stability and purity/homogeneity (responses 6–8). There also were questions about the detoxification products—how to detect them and whether the treated products would be palatable (responses 10 and 14).

MyToolBox and its successors could enhance ongoing work on detoxification of mycotoxin-contaminated feed materials in China (Table 10) with binders and enzymes for detoxification processes. Concerns were expressed about both chemical products produced by detoxification and the detoxified products themselves. Several responses were ambiguous and could be applied to a detoxification agent/process or to a detoxified product. The top two responses on methodology, procedures, and standard operating protocols (highlighting the need for both certified reference materials and international proficiency testing schemes) were both ambiguous and could apply to the process for making the detoxification agent or to the detoxified product (responses 1 and 2). There is a need for more information on the detoxification process reflected in the requests for additional training (responses 4, 5, 10 and 11), and in the requests for accessible information from reliable sources (responses 3 and 9). Satisfying these needs could be done through a platform that enabled multiple China/EU collaborations and included local workshops and training (response 12). Remaining responses concentrated on the detoxified product—evaluation for safety, suitability for consumption by a target species (potentially as both food and feed), and standards for use in detection of/screening for detoxified products (responses 6–8).

### 2.6. Safe Use Option

Responses provided to the question “What are the current issues and ongoing research on Safe Use Options of mycotoxin-contaminated materials in China?” included (Table 10): (i) cost and economic viability of the safe use option approach, (ii) safety, efficacy and stability of detoxification products (mycotoxin degrading enzymes), (iii) identification of conditions under which detoxification products can be used, and (iv) potential uses of detoxified materials.

Responses to the question “How can MyToolBox (or successor collaborative projects) enhance ongoing work on detoxification of mycotoxin-contaminated materials in China?” were quite general. Most fall under a broad rubric of “Provide guidance for the safe use option approach” with requests for shared methods and procedures, additional training for personnel, and common safety, production and detection protocols. Beyond this request for guidance, participants wanted to create awareness of the mycotoxin problem beyond the MyToolBox community and to ensure that the MyToolBox e-platform is freely available in Chinese, both on and off line and that the e-platform included the experience of universities, research institutes and industries with safe use protocols.

## 3. Discussion

### 3.1. Biocontrol

Climate change is resulting in more extreme weather events in many parts of the world, with increased heat and drought two examples of ways that these changes stress crop plants. Changes in both the EU and China increased the geographic range where aflatoxin contamination can occur and intensification of the problem in areas where contamination already occurs. Within the MyToolBox project, an atoxigenic *Aspergillus flavus* strain, Af01, which is native to Serbia, was used as a biocontrol agent in irrigated and non-irrigated maize fields in Serbia during the 2016 and 2017 cropping seasons. This biocontrol treatment reduced aflatoxin contamination, on average, by 73% (range 51–83%). Thus, aflatoxin contamination of maize in Serbia can be reduced through biological control with a native atoxigenic *A. flavus* strain [27].

To comply with the Nagoya protocol [28] on biodiversity and to limit the spread of introduced strains of *Aspergillus*, Chinese workers are following a parallel process to develop local atoxigenic strains of *A. flavus* for use in China on maize and peanuts. There is high demand in China for a sustainable pre-harvest method that reduces or eliminates aflatoxin contamination. Follow-up issues include: (i) how to apply the biocontrol agents in a commercial setting, (ii) identifying microorganisms responsible for degradation of mycotoxins, and (iii) creating a standard process for continuous development of new biocontrol agents in China. Stakeholders were very interested in future EU–China collaborations to develop and implement biocontrol agents for various regions in China, and to maintain and update control methods, possibly by using the MyToolBox e-platform. Biocontrol research currently is riding a wave of scientific popularity with the success of AflaSafe [29] for the reduction of aflatoxins in maize and peanuts in sub-Saharan Africa [30]. This product can be easily adapted to or developed for particular in-country situations [31].

### 3.2. Forecasting

Forecasting has become a much more prominent issue with multiple forecasting models now available for multiple toxins in multiple crops [21,32,33,34,35,36,37,38,39]. Although some models incorporate only weather variables, most also include farm-specific agronomic information and geographic location. Some of the models are empirical, i.e., defined by relationships between input and output data, but others are mechanistic, i.e., are based on the biology of the toxin-producing fungi growing under particular conditions. The existing models are not perfect, but several can give 80% or more accuracy in locations to which they have been adapted [21,34]. MyToolBox has various forecasting models available in the dynamic part of the e-tool. These models were improved and validated with real data obtained during the project.

The Chinese stakeholders were very interested in the mycotoxin forecasting models, their development, their implementation, and especially in using machine learning in combination with big data. The need to transform both analytical data and field survey data to usable information for farmers and researchers remains to be addressed. Data sharing for supply chain analysis, tailored model development for Chinese end-users, and the cost-effectiveness of forecasting models/DSSs are important areas of interest for future EU–China collaborations.

### 3.3. Sampling and Analysis

Sampling and analysis protocols are key to estimating risks and enforcing food safety and phytosanitary requirements. The irregular distribution of mycotoxins within a sample can make obtaining a representative sample difficult. In the European Union, sampling and analysis for official sampling and control of (multiple) mycotoxins is regulated by Regulation (EC) No. 401/2006 [40] and 2014/519 [41], setting requirements for the method’s precision, repeatability and reproducibility (amongst others)for different foods. In recent years significant advances have been made to increase the speed of the analysis, lower the limits of detection and increase the number of compounds that can be measured. A MyToolBox goal was to identify cost-effective methods for required sampling and analysis, e.g., for routine screening or enforcing legislation [42]. The most commonly used analytical methods for the determination of various mycotoxins simultaneously are based on liquid chromatography coupled with mass-spectrometry (LC-MS/MS) [43]. In case of non-official controls for the onsite detection of a limited number of mycotoxins, enzyme-linked immunosorbent assays (ELISA), e.g., for aflatoxin B1 in maize or deoxynivalenol in wheat, or lateral flow devices (LFD) for deoxynivalenol in wheat, might provide quicker or more cost-effective alternatives [42]. In Europe, ELISA-based analysis for screening was slightly more effective than LC-MS/MS or LFD-based methods when analyzing wheat batches for deoxynivalenol, with differences in accuracy for deoxynivalenol contamination in wheat found to be minimal [42]. Surprisingly, on-site detection with LFDs turned out to be the least cost-effective method [43].

Sampling and analysis were of great interest to the Chinese stakeholders, and this group had more stakeholders present than any other. All aspects of sampling and analysis had the stakeholders’ attention—starting from ensuring high quality samples were collected by properly trained personnel, to transport to the analytical facilities, and subsequent high quality analyses of the materials collected. Many stakeholders were interested in collaborating with MyToolBox and its successors through the exchange of harmonized analytical methods, increased availability of analytical standards, and the use of rapid screening methods to make initial evaluations of mycotoxin contamination on-site. Finally, there was interest in collaboration on methods for tracking and tracing samples with the associated batches of raw materials, food or feed from which the samples were taken.

### 3.4. Silo Management

Chinese stakeholders expressed interest in effective post-harvest management of staple foodstuffs especially rice, maize, wheat and peanuts to minimize both losses and export rejections due to mycotoxin contamination. Regional differences in the quality of the harvested product and silo design and management require tailor-made solutions rather than generic approaches to solving problems. At present, short-term sensing systems for the key abiotic factors such as relative humidity (RH), temperature or CO_2_ are used in Chinese storage facilities, and development of permanent sensors and models to use the data these sensors report has been an important step forward. A real time Decision Support System (DSS) was developed in the MyToolBox project which can be used in conjunction with biological models for marginal and optimum abiotic conditions for the initiation of fungal growth and potential contamination of stored wheat with zearalenone or ochratoxin A or of stored maize or peanuts with aflatoxins. Once changes in CO_2_ levels in real time inside a silo could be visualized it was possible to identify regions within a silo where fungal/insect respiration is occurring and where stored materials can be contaminated by mycotoxins. MyToolBox partners from China and UK have worked together closely on-site at Chinese storage facilities to improve post-harvest management by implementing effective remedial actions, e.g., aeration or removal of potentially contaminated material from the silo, based on trial versions of the DSS.

Management of silos and other post-harvest issues are critical for managing losses of food after it has been produced. Moving materials from farm gate to storage and preparation of harvested crops prior to storage can significantly affect the time the crop can be stored and the quality of the material that is taken from storage. In principle, the most important thing to do is to reduce moisture content below a fixed threshold (varies by crop). China has a range of post-harvest practices and storage facilities that are rapidly being modernized and that require sophisticated trained managers. The form of these storage structures can be uniquely Chinese and practical management solutions may be unique as well, even though the problems being addressed are similar to those encountered elsewhere.

### 3.5. Detoxification

With increased surveillance and lower limits of allowable contamination, the amount of material that could potentially need detoxification is increasing.

Feed additives for mycotoxin detoxification are substances which, when incorporated in animal feed, either bind mycotoxins so they are no longer bioavailable or act as bio-transforming agents converting the toxins into less biologically active products. The scientific literature covering mycotoxin detoxification in animal feed is large and numerous substances have been promoted either as physical or biological adsorbents, or as microbiological/enzymatic transformation agents. Particularly exhaustive is the 2009 report commissioned by European Food Safety Authority (EFSA) to review mycotoxin-detoxifying agents used as feed additives, their mode of action, efficacy and feed/food safety [44].

Feeding trials are crucial to evaluate the efficacy of mycotoxin detoxifying feed additives in target species. In the last decade, the focus of these in vivo studies has shifted from the sole assessment of performance data to the evaluation of specific mycotoxin biomarkers in biological matrices of exposed animals. The biomarker data enable demonstration of increased excretion/reduced deposition of mycotoxins, which is an important criterion for registration of mycotoxin-detoxifying feed additives in the EU.

The efficacy of feed additives to reduce the absorption of dietary aflatoxin B1 in dairy cows and absorption of dietary fumonisin B1 in pigs were investigated through in vivo experiments conducted at the Feed Research Institute of the Chinese Academy of Agricultural Sciences CAAS (CAAS-FRI). Both studies were undertaken in China and used EU mycotoxin detoxification additives for aflatoxins in dairy cows and for fumonisins in pigs. MyToolBox partners successfully tested the efficacy of these feed additives under local conditions in China according to EC Regulation No. 429/2008 for the evaluation of feed additives. This test was an unprecedented step forward in establishing common scientific (and ethical) bases for feeding trials jointly recognized by the EU and China [20].

Globalization implies increased import–export exchanges combined with different, and decreasing, allowable contamination levels. Thus, post-harvest practices that minimize mycotoxin contamination remain important considerations, a point that also was reflected in the round table discussions. In addition to effective chemical–physical treatments, e.g., cleaning, sorting, roasting, binding agents or ammoniation/chlorination/ozonation, there was growing interest in enzymatic treatments targeted towards particular toxins. Lack of familiarity with many of these processes led to questions about the properties and results of these treatments. Future Chinese cooperation with MyToolBox and its successors should include developing common operational protocols and identifying active agents, reference materials, and analytical standards to monitor safety of the treated end products.

### 3.6. Safe Use Option

When the topics for the round table discussion sessions were established there was no clear differentiation between the topics of “detoxification” and “safe use options”. Detoxification focuses on animal feed additives, while the focus of safe use options is on detoxification technologies outside of animal feed, e.g., enzymes used in biofuel production. Both topics were included in this session which led to overlap in topic identification and goal setting between the two topics. In the MyToolBox context the use of binders falls in the detoxification category. The use of mycotoxin binders that prevent absorption of toxins in the gut of animals that consume contaminated feed is an important “detoxification” option. The “safe use option” in the MyToolBox context defines the application of mycotoxin degrading enzymes, which were developed and registered for the use in animal feed, in processes such as bioethanol and biogas production. During bioethanol production, mycotoxin concentration increases by a factor of three on a dry weight basis in DDGS compared with the starting grain [45]. The use of mycotoxin detoxifying enzymes in the fermentation process increases valorization of DDGS as a feed ingredient, because grain batches with mycotoxin levels that exceed limit and guidance values can have these values greatly reduced even while using the contaminated grain is used as a substrate for biofuel production. Alternatively, highly contaminated grain can be used as a feedstock for bioethanol production to generate biogas as an energy source.

When strategies to produce raw materials with low levels of mycotoxin contamination have failed, as have post-harvest strategies, such as proper storage, cleaning and sorting or adding binders, the remaining materials are unfit for use as food or feed. Instead of destruction, the contaminated material could be used for bioethanol production, but high levels of contaminated material may reduce process efficiency and result in DDGS with too much mycotoxin to be used as animal feed. MyToolBox partners adapted mycotoxin degrading enzymes to the bioethanol process to increase fermentation efficiency and produce DDGS with mycotoxin levels below legal limits for use in animal feed.

## 4. Conclusions

The EU–China cooperation carried out within the MyToolBox project and the discussions held at the recent stakeholder workshop have contributed to a better understanding of the scientific, technological, economic but also social challenges the EU and China are facing within their joint effort to develop smart and effective tools and strategies to control and mitigate the mycotoxin issues. The development of a novel decision support system in post-harvest silo management research and the performance of feeding trials in China to test the efficacy of binders and recombinant enzymes as detoxifiers according to EU regulations under local conditions in China can be considered as the most prominent achievements of MyToolBox. On the other hand, the unique workshop held in Beijing clearly demonstrated that there is still a great need for smart and integrated strategies to tackle the mycotoxin issue to achieve safer food and feed products and to minimize losses and export rejections.

## 5. Methods

Roundtable discussions were conducted with a moderated discussion technique, the Nominal Group technique. The technique enables equal input from all participants and is useful for generating a large number of ideas, while also providing a mechanism for ranking them. The list of ideas provides a rich and detailed context from which novel ideas and general trends can be extracted. During the workshop in Beijing each of the six groups was asked to consider two questions: (i) What are the current issues and ongoing research in their topic area in China? and (ii) How can MyToolBox (or successor collaborative projects) enhance ongoing work in their topic area in China?

Nominal Group discussions usually divide participants into small groups of 5–8 members, preferably multi-disciplinary in nature, led by a moderator and reporter (Table 11). All the stakeholders involved in the roundtable discussion were from China. EU participants were only facilitators of the discussion. Normally, these groups stay together through multiple sessions over the course of the conference, and multiple groups receive the same questions, and answers common to multiple groups are rated as more important than those limited to just a single group. For practical reasons, at this workshop, there were three groups for discussions on each of two days reflecting the three topics discussed at the conference that day. Thus, comparisons between groups to help identify more prominent ideas was not possible. Groups were all (15–50 persons in size) larger than the preferred maximum of 10, and not all members of a group submitted responses to the questions. All responses are presented here in English, even though many were originally submitted in Chinese.

**Figure 1:**
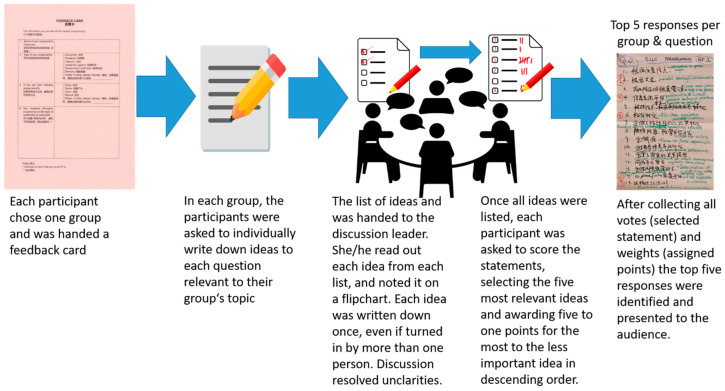
Schematic outline of the Nominal Group Style roundtable discussion process.

The general format for the discussion of each question (Figure 1) was:

1. Silent generation of ideas. Each individual considers responses to the question and writes them down on a sheet of paper to facilitate the subsequent discussion. Participants were not to talk with one another during this time so that the ideas generated are their own and not part of a larger group thinking process. The goal was to generate as many ideas as possible, and not to arrive at a consensus. Answers could be provided in English or Chinese, and individuals were encouraged to submit multiple responses to a question.
2. Sharing ideas. The reporter and the moderator wrote responses on a flip chart and could shorten them to a word or a phrase that captured the essence of the response. A brief discussion of each item followed to ensure that the meaning of the idea/word/phrase was understood, but not to discuss the importance of the idea/word/phrase. There was no expectation that each member of the group would provide the same number of responses. A common target for these lists is 15–25 unique responses per question. Irrelevant and duplicative answers were purged from the lists as well as most items that received no votes, so the number of items in a table may be less than 15.
3. Voting and ranking. Once the ideas/words/phrases were adequately explained, each participant ranked the five most important responses, with the most important response being given a “5”. The second choice answer received a “4”, the next a “3”, and so on. This process was done silently as the goal was to record individual preferences and not to reach a consensus, even though a consensus may be observed when the numbers are tallied. Individual ranks were recorded next to each response on a flip chart. The flip chart paper and the individual sheets with rankings were turned into the session organizers for further analysis.
4. Presentation of results. The five highest ranked responses were reported at the plenary session that followed the group discussions. A complete listing of responses and commentary on the responses are summarized in Results (Table 1, Table 2, Table 3, Table 4, Table 5, Table 6, Table 7, Table 8, Table 9 and Table 10).

## Figures and Tables

**Table 1:** Group discussion responses to question: What are the current issues and ongoing research in biocontrol of mycotoxin contamination in China?

	*^x^*#	*^y^S*	Response
1	14	37	Cost to use biological control agents commercially
2	12	39	Toxin degradation in various cereals
3	12	37	How to evaluate the effect of biological control agents in the actual production
4	10	40	Are biological control agents safe?
5	8	23	Degradation of mycotoxins by microorganisms
6	8	20	How to identify non-toxigenic strains
7	5	15	Which biological control technologies are mature and ready for use?
8	4	10	Recovery of biological control agents from soil
9	3	7	Application of biological control methods to the cultivation of Chinese herbal medicines

*^x^*#—Number of participants ranking this response as one of the five most important. *^y^S*—Weighted priority score, with each participant ranking their top five topics. Five points are assigned to the most important response and one point to the least significant of the important responses.

**Table 2:** Group discussion responses to: How can MyToolBox (or successor collaborative projects) enhance ongoing work in biocontrol of mycotoxin contamination in China?

	*^x^*#	*^y^S*	Response
1	12	38	Impacts of climate change and drug resistance on biological control
2	12	38	Increase development and cooperation with Chinese institutions working on biocontrol
3	11	31	Establish a cooperative platform for mycotoxin research between Europe and China
4	9	31	Promotion of MyToolBox technologies for use on small farms in China
5	7	26	Other forms of application of MyToolBox
6	7	25	Research on the prevention and control of additional mycotoxin-producing fungi
7	7	19	Integration and collaboration of research on mycotoxins to solve common global problems
8	6	19	Will MyToolBox software be publicly accessible? Will there be a fee to use it?
9	6	17	Provide more pre-trial opportunities

*^x^*#—Number of participants ranking this response as one of the five most important. *^y^S*—Weighted priority score, with each participant ranking their top five topics. Five points are assigned to the most important response and one point to the least significant of the important responses.

**Table 3:** Group discussion responses to: What are the current issues and ongoing research in forecasting of mycotoxin contamination in China?

	*^y^S*	Response
1	5	Identified roles for university/governmental and private sector entities/enterprises in early warning system
2	3.5	Production recommendations based on forecasting system results and data
3	3.5	Establish an identification and early warning system for entire production chains
4	2	Information collection, disclosure, and sharing protocols
5	1	Research on early warning technology
6	•	Mycotoxin regulations are not sufficiently comprehensive
7	•	Mycotoxin contamination risk/history maps
8	•	Development of awareness of risks posed by mycotoxin contamination
9	•	Application of big data concepts and technologies

*^y^S*—Weighted composite priority score for the top five responses, based on each participant ranking their top five topics. Five indicates the most important response and one the least significant of the ranked responses. Unranked responses are not listed in any particular order.

**Table 4:** Group discussion responses to: How can MyToolBox (or successor collaborative projects) enhance ongoing work in forecasting of mycotoxin contamination in China?

	*^y^S*	Response
1	5	Establish a data sharing system
2	4	Pay attention to particular farms and enterprises
3	3	Expand early warning range of crops and toxins
4	2	Consideration of Chinese characteristics and conditions
5	1	Incorporate an economic factor in the early warning system
6	•	Participation of large companies (to provide more data)
7	•	Increase MyToolbox promotion in China by setting up an official contact for critical services, e.g., providing information on the use of MyToolBox models
8	•	Compare multiple modeling methods
9	•	Consider potential biological/chemical protection in early warning models
10	•	Focus on the production chain of models beyond in-field growth
11	•	Official promotion of early warning model application
12	•	Identify impacts of climate change based on early warning model results

*^y^S*—Weighted composite priority score for the top five responses, based on each participant ranking their top five topics. Five indicates the most important response and one the least significant of the ranked responses. Unranked responses are not listed in any particular order.

**Table 5:** Group discussion responses to: What are the current issues and ongoing research in sampling and analysis of mycotoxin contamination in China?

	^x^#	*^y^S*	Response
1	14	43	Representative means and standard deviations of test results
2	14	41	Requirements for and training of samplers
3	13	38	Low-cost, effective sampling methods
4	10	23	LODs and LOQs of standard instruments and protocols
5	9	32	Simple, effective sample pre-treatment methods
6	8	29	Optimal conditions for sample transfer and storage across the value chain
7	6	15	Availability of chemical standards of mycotoxins and degradation products
8	5	18	Uniformity and representativeness of batch samples
9	5	16	Quick check method
10	5	16	Representative sampling from a large number of raw materials in bulk
11	5	10	Minimization of deviations caused by inspectors
12	4	8	Interference with sample integrity after detoxification
13	1	5	Processing representative sample during preparation and detection
14	1	2	Simple and effective matrix specific methods

*^x^*#—Number of participants ranking this response as one of the five most important. *^y^S*—Weighted priority score, with each participant ranking their top five topics. Five points are assigned to the most important response and one point to the least significant of the important responses.

**Table 6:** Group discussion responses to: How can MyToolBox (or successor collaborative projects) enhance ongoing work in sampling and analysis of mycotoxin contamination in China?

	*^x^*#	*^y^S*	Response
1	14	46	Can source-to-product traceability be provided by MyToolBox?
2	9	33	Recommendations for sampling methods
3	8	25	Require detailed standard plans for sampling and testing
4	7	19	Develop sample matrix control strategies
5	7	18	Online quick test
6	7	16	Online assessment and monitoring program
7	6	24	How to assess risks posed by materials from different geographic regions
8	6	17	Assessment of contamination in different maize varieties/lines/hybrids
9	6	16	Facilitate MyToolBox cooperation with companies?
10	5	19	Free sharing of sampling models
11	4	15	Can MyToolBox enable the mailing of samples between Europe and China
12	3	10	Monitoring programs in specific regions with high risks

*^x^*#—Number of participants ranking this response as one of the five most important. *^y^S*—Weighted priority score, with each participant ranking their top five topics. Five points are assigned to the most important response and one point to the least significant of the important responses.

**Table 7:** Group discussion responses to: What are the current issues and ongoing research in silo/post-harvest management to reduce mycotoxin contamination in China?

	*^x^*#	*^y^S*	Response
1	12	44	Monitoring, forecasting models and processing of data on the status of stored materials
2	9	31	Representative sampling and sampling costs
3	9	27	Sensors
4	9	25	Cost/usage of drying and related energy issues
5	9	20	Standardization of silo storage techniques
6	7	28	Control mycotoxin contamination
7	7	20	Application of regional model for silos
8	5	14	Regional differences in grain quality and the ability to deal with it
9	4	13	Insect damage
10	4	10	Pre-storage sorting
11	3	11	Infrastructure for spraying fungicides
12	3	6	Maintaining food safety during transport
13	3	5	Water migration in large silos

*^x^*#—Number of participants ranking this response as one of the five most important. *^y^S*—Weighted priority score, with each participant ranking their top five topics. Five points are assigned to the most important response and one point to the least significant of the important responses.

**Table 8:** Group discussion responses to: How can MyToolBox (or successor collaborative projects) enhance ongoing work on silo/post-harvest management to reduce mycotoxin contamination in China?

	*^x^*#	*^y^S*	Response
1	13	39	Model (e.g., MyToolBox) sharing, collaboration and integration with existing apps
2	12	40	Free e-platform that enables information, knowledge and data exchange
3	8	23	Monitoring during transportation
4	7	21	Decision support system (DSS) for management of different types of silos
5	6	14	Early warning risk assessment system
6	5	15	EU–China mycotoxin regulation harmonization
7	8	24	Information on grain origin management of material from high-risk areas
8	4	10	Model development relating insect damage and mycotoxin formation
9	4	10	Farm to Fork approach
10	3	12	Training on mycotoxin analysis
11	1	2	Provide advice based on analytical results

*^x^*#—Number of participants ranking this response as one of the five most important. *^y^S*—Weighted priority score, with each participant ranking their top five topics. Five points are assigned to the most important response and one point to the least significant of the important responses.

**Table 9:** Group discussion responses to: What are the current issues and ongoing research in detoxification of mycotoxin-contaminated materials in China?

	*^x^*#	*^y^S*	Response
1	10	45	The safety of detoxification
2	8	25	The cost and profits of detoxification agents? Return on investment?
3	8	19	Application conditions and time required for detoxification agents to work
4	7	16	The evaluation process for detoxification enzymes
5	5	13	Efficacy of detoxification processes
6	5	9	Evaluation of mycotoxin adsorbents on adsorption of nutrients
7	4	10	Stability of detoxification agents
8	4	13	Homogeneity and purity of detoxification agents
9	3	10	High-speed and efficient method for screening bacteria that produce enzymes
10	3	9	Detection of compounds resulting from detoxification processes
11	3	8	Is use of detoxification enzymes in feed allowed in China?
12	2	7	Application scope for detoxification agents
13	2	8	Identify companies that produce detoxification enzymes
14	1	3	Palatability of detoxification agents

*^x^*#—Number of participants ranking this response as one of the five most important. *^y^S*—Weighted priority score, with each participant ranking their top five topics. Five points are assigned to the most important response and one point to the least significant of the important responses.

**Table 10:** Group discussion responses to: How can MyToolBox (or successor collaborative projects) enhance ongoing work in detoxification of mycotoxin-contaminated materials in China?

	*^x^*#	*^y^S*	Response
1	11	43	Sharing methods and procedures
2	9	27	Can MyToolBox develop standardized operating techniques for production?
3	8	22	Updates (blog?) on research and information on current detoxification research
4	5	18	Technical and personnel training
5	5	10	More meetings, conference and seminars for discussion and learning
6	4	14	Optimize the conformity of product and animal species
7	4	10	Shared methods for evaluating safety
8	4	10	Standards for detection of detoxification products
9	4	8	Build a database of potential detoxification products
10	3	12	Training for MyToolBox users
11	2	3	Demonstration of detection methods for detoxified products
12	1	3	Platform that enables multiple China/EU collaborations

*^x^*#—Number of participants ranking this response as one of the five most important. *^y^S*—Weighted priority score, with each participant ranking their top five topics. Five points are assigned to the most important response and one point to the least significant of the important responses.

**Table 11:** Discussion group leader of the “round table discussion”.

Session	Discussion Group Leaders
Biocontrol	Ferenc Bagi ^1^
	Yang Liu ^2^
	Changpo Sun ^3^
Sampling and Analysis	John Gilbert ^4^
	Monique de Nijs ^5^
	David Zhang ^6^
Forecasting	Cheng Liu ^5^
	Songxue Wang ^3^
Silo Management	Songxue Wang ^3^
	Zhongjie Zhang ^3^
	Naresh Magan ^7^
Detoxification	Jinquan Wang ^8^
	Qingwei Wang ^9^
	John Gilbert ^4^
	Michele Suman ^10^
Safe Use Options	Gerd Schatzmayr ^11^
	Michael Klingeberg ^12^
	Guangtao Zhang ^13^

^1^ University of Novi Sad, Faculty of Agriculture, Dositeja Obradovica 8, Novi Sad, Serbia; ^2^ Institute of Agro-Products Processing Science and Technology, Chinese Academy of Agricultural Sciences, No. 2 Yuanmingyuan West Road, Haidian District, Beijing, China; ^3^ Academy of National Food and Strategic Reserves Administration, Nr. 11 Baiwanzhuang Street, Beijing, China; ^4^ FoodLife International Ltd., Ara-1 Çankaya Ankara, Turkey; ^5^ Wageningen Food Safety Research, Akkermaalsbos 2, Wageningen, The Netherlands; ^6^ Romer Labs China Ltd., Room 1413, Building A, Jia Tai International Mansion 41 East 4th Ring Middle Road Chaoyang District, Beijing, China; ^7^ Cranfield University, College Road, Cranfield, Bedfordshire, Great Britain; ^8^ Feed Research Institute, Chinese Academy of Agricultural Sciences (CAAS-FRI), No. 2 Yuanmingyuan West Road, Haidian District, Beijing, China; ^9^ Biomin China, No. 6-1 Chunyu Road, Xishan Economic and Technological Development Zone, Wuxi, China; ^10^ BARILLA S.p.A., Food Chemistry and Safety Research, Barilla Research Labs, Italy; ^11^ BIOMIN Research Center, BIOMIN Holding GmbH, Technopark 1, Tulln, Austria; ^12^ Südzucker Germany AG, Post box 1127, Wormser Straße 11, Obrigheim, Germany; ^13^ Mars Global Food Safety Center, 2 Yanqi East Second Street, Huairou District, Beijing, China.

## References

[1] Eskola M., Kos G., Elliott C.T., Hajšlová J., Mayar S., Krska R. (2019). Worldwide contamination of food-crops with mycotoxins: Validity of the widely cited ‘FAO estimate’ of 25%. Crit. Rev. Food. Sci. Nutr..

[2] Cimbalo A., Alonso-Garrido M., Font G., Manyes L. (2020). Toxicity of mycotoxins in vivo on vertebrate organisms: A review. Food Chem. Toxicol.

[3] Da Rocha M.E.B., Freire F.D.O., Maia F.B.F., Guedes M.I.F., Rodina D. (2014). Mycotoxins and their effects on human and animal health. Food Control..

[4] Van Egmond H.P., DeVries J.W., Trucksess M.W., Jackson L.S. (2008). Worldwide regulation for mycotoxins. Mycotoxin and Food Safety, Part of the Advances in Experimental Medicine and Biology Book Series.

[5] Medina A., Baazeem A.A.A., Rodriguez A., Magan N. (2017). Climate change, food security and mycotoxins: Do we know enough?. Fungal Biol. Rev..

[6] MyToolBox The Smart Way to Tackle Mycotoxins. EU-Funded Project within Horizon 2020, 2016–2020. www.mytoolbox.eu.

[7] MycoKey Integrated and Innovative Key Actions for Mycotoxin Management in the Food and Feed Chains. EU-Funded Project within Horizon 2020, 2016–2020. www.mycokey.eu.

[8] Krska R., de Nijs M., McNerney O., Pichler M., Gilbert J., Edwards S., Suman M., Magan N., Rossi V., van der Fels-Klerx H.J. (2016). Safe food and feed through an integrated toolbox for mycotoxin management: The MyToolBox approach. World Mycotoxin J..

[9] E-Platform of MyToolBox The Smart Way to Tackle Mycotoxins. EU-Funded Project within Horizon 2020, 2016–2020. https://mytoolbox-platform.com.

[10] Gruber-Dorninger C., Jenkins T., Schatzmayr G. (2019). Global mycotoxin occurrence in feed: A ten-year survey. Toxins.

[11] Eskola M., Elliott C.T., Hajšlová J., Steiner D., Krska R. (2020). Towards a dietary-exposome assessment of chemicals in food: An update on the chronic health risks for the European consumer. Crit. Rev. Food Sci. Nutr..

[12] RASFF. https://webgate.ec.europa.eu/rasff-window/portal/.

[13] Zhang W.W., Ye Z.M., Jin Y., Wang S.Y., Zhang L.S., Pei Zhang X.F. (2014). Management of mycotoxin contamination in food and feed in China. World Mycotoxin J..

[14] USDA (United States Department of Agriculture) China Releases Standards for Maximum Levels of Mycotoxins in Foods. 22 June 2018. https://www.fas.usda.gov/data/china-china-releases-standard-maximum-levels-mycotoxins-foods.

[15] Janssen E.M., Mourits M.C.M., van der Fels-Klerx H.J., Oude Lansink A.G.J.M. (2019). Pre-harvest measures against *Fusarium* spp. Infection and related mycotoxins implemented by Dutch wheat farmers. Crop. Prot..

[16] Garcia-Cela E., Kiaitsi E., Sulyok M., Medina A., Magan N. (2018). *Fusarium graminearum* in stored wheat: Use of CO₂ production to quantify dry matter losses and relate this to relative risks of zearalenone contamination under interacting environmental conditions. Toxins.

[17] Garcia-Cela E., Kiaitsi E., Sulyok M., Krska R., Medina A., Petit Damico I., Magan N. (2019). Influence of storage environment on maize grain: CO_2_ production, dry matter losses and aflatoxins contamination. Food Addit Contam Part A.

[18] Garcia-Cela E., Cela F., Gari F.J., Sanchez C., Sulyok M., Verheecke-Vaessen C., Medina A., Krska R., Vaessen N. (2020). Carbon dioxide production as an indicator of *Aspergillus flavus* colonization and aflatoxin/cyclopiazonic acid contamination in shelled peanuts stored under different interacting abiotic factors. Fungal Biol..

[19] Stadler D., Lambertini F., Bueschl C., Wiesenberger G., Hametner C., Schwartz-Zimmermann H., Hellinger R., Sulyok M., Lemmens M., Schuhmacher R. (2019). Untargeted LC–MS based 13C labelling provides a full mass balance of deoxynivalenol and its degradation products formed during baking of crackers, biscuits and bread. Food Chem..

[20] Krska R. Setting Feed Additive Standards: An EU and China Comparison. All About Feed, October 2019. https://www.allaboutfeed.net/Mycotoxins/Articles/2019/10/Setting-feed-additive-standards-An-EU-and-China-comparison-479814E/.

[21] Liu C., Manstretta V., Rossi V., van der Fels-Klerx H.J. (2018). Comparison of three modeling approaches for predicting deoxynivalenol contamination in winter wheat. Toxins.

[22] Delbecq A.L., van de Ven A.H., Gustafson D.H. (1975). Group Techniques for Program Planning: A Guide to Nominal Group and Delphi Processes.

[23] Leslie J.F., Lattanzio V., Audenaert K., Battilani P., Cary J., Chulze S.N., De Saeger S., Gerardino A., Karlovsky P., Liao Y.-C. (2018). MycoKey round table discussions on future directions in research on chemical detection methods, genetics and biodiversity of mycotoxins. Toxins.

[24] Bandyopadhyay R., Frederiksen R.A., Leslie J.F., Leslie J.F., Bandyopadhyay R., Visconti A. (2008). Priorities for mycotoxin research in Africa identified by using the nominal group technique. Mycotoxins: Detection Methods, Management, Public Health and Agricultural Trade.

[25] Leslie J.F., Frederiksen R.A., Leslie J.F., Frederiksen R.A. (1995). Variable pathogens: The changing scenario. Disease Analysis through Genetics and Biotechnology: Interdisciplinary Bridges to Improved Sorghum and Millet Crops.

[26] Vanhoutte I., Audenaert K., de Gelder L. (2016). Biodegradation of mycotoxins: Tales from known and unexplored worlds. Front. Microbiol..

[27] Savić Z., Dudaš T., Loc M., Grahovac M., Budakov D., Jajić I., Krstović S., Barošević T., Krska R., Sulyok M. (2020). Biological Control of Aflatoxin in Maize Grown in Serbia. Toxins.

[28] Convention on Biological Diversity. https://www.cbd.int/abs/.

[29] Aflasafe^®^, Safer Food in Africa. https://aflasafe.com.

[30] Bandyopadhyay R., Ortega-Beltran A., Akande A., Mutegi C., Atehnkeng J., Kaptoge L., Senghor L.A., Adhikari B.N., Cotty P.J. (2016). Biological control of aflatoxins in Africa: Current status and potential challenges in the face of climate change. World Mycotoxin J..

[31] Senghor L.A., Ortega-Beltran A., Atehnkeng J., Callicott K.A., Cotty P.J., Bandyopadhyay R. (2019). The atoxigenic biocontrol product AflaSafe SN01 is a valuable tool to mitigate aflatoxin contamination of both maize and groundnut cultivated in Senegal. Plant. Dis..

[32] Battilani P., Camardo Leggieri M. (2015). Predictive modelling of aflatoxin contamination to support maize chain management. World Mycotoxin J..

[33] Battilani P., Toscano P., van der Fels-Klerx H.J., Moretti A., Camardo Leggieri M., Brera C., Rortais A., Goumperis T., Robinson T. (2016). Aflatoxin B1 contamination in maize in Europe increases due to climate change. Sci. Rep..

[34] De Wolf E., Paul P.A., Leslie J.F., Logrieco A.F. (2014). Predicting mycotoxin contamination in wheat. Mycotoxin Reduction in Grain Chains.

[35] Birr T., Verreet J.A., Klink H. (2019). Prediction of deoxynivalenol and zearalenone in winter wheat grain in a maize-free crop rotation based on cultivar susceptibility and meteorological factors. J. Plant. Dis. Protect..

[36] Nazari L., Manstretta V., Rossi V. (2016). A non-linear model for temperature-dependent sporulation and T-2 and HT-2 production of *Fusarium langsethiae* and *Fusarium sporotrichioides*. Fungal Biol..

[37] Sancho A.M., Moschini R.C., Filippini S., Rojas D., Ricca A. (2018). Weather-based logistic models to estimate total fumonisin levels in maize kernels at export terminals in Argentina. Trop. Plant. Pathol..

[38] Van der Fels-Klerx H.J., Dekkers S., Kandhai M.C., de Heer C. (2010). Indicators for early indetification of re-emerging mycotoxins. Njas-Wagen. J. Life Sci..

[39] Van der Fels-Klerx H.J., Goedhart P.W., Elen O., Borjesson T., Hietaniemi V., Booij C.J.H. (2012). Modeling deoxynivalenol contamination of wheat of northwestern Europe for climate change assessment. J. Food Protec..

[40] Commission of the European Communities (2006). Commission Regulation (EC) No 401/2006 of 23 February 2006 laying Down the Methods of Sampling and Analysis for the Official Control of the Levels of Mycotoxins in Foodstuffs (Text with EEA Relevance). OJL.

[41] European Commission (2014). Commission Regulation (EU) No 519/2014 of 16 May 2014 amending Regulation (EC) No 401/2006 as regards methods of sampling of large lots, spices and food supplements, performance criteria for T-2, HT-2 toxin and citrinin and screening methods of analysis (Text with EEA relevance). OJL.

[42] Focker M., van der Fels-Klerx H.J., Oude Lansink A.G.J.M. (2018). Cost-effective sampling and analysis for mycotoxins in a cereal batch. Risk Anal..

[43] Krska R., Sulyok M., Berthiller F., Schuhmacher R. (2017). Mycotoxin testing: From multi-toxin analysis to metabolomics. SJM Mycotoxins.

[44] EFSA (2019). Review of Mycotoxin-Detoxifying Agents used as Feed Additives: Mode of Action, Efficacy and Feed/Food Safety. https://www.efsa.europa.eu/en/supporting/pub/en-22.

[45] Schaafsma A.W., Rios V.L., Rios Paul D.E., Miller J.D. (2009). Mycotoxins in fuel ethanol co-products derived from maize: A mass balance for deoxynivalenol. J. Sci. Food Agric..

